# Nanostructured lipid carriers containing benznidazole: physicochemical, biopharmaceutical and cellular in vitro studies

**DOI:** 10.3762/bjnano.14.66

**Published:** 2023-07-28

**Authors:** Giuliana Muraca, María Esperanza Ruiz, Rocío C Gambaro, Sebastián Scioli-Montoto, María Laura Sbaraglini, Gisel Padula, José Sebastián Cisneros, Cecilia Yamil Chain, Vera A Álvarez, Cristián Huck-Iriart, Guillermo R Castro, María Belén Piñero, Matias Ildebrando Marchetto, Catalina Alba Soto, Germán A Islan, Alan Talevi

**Affiliations:** 1 See end of main text.

**Keywords:** benznidazole, biopharmaceutical study, Chagas disease, nanoparticles, nanostructured lipid carriers, physicochemical characterization, *Trypanosoma cruzi*

## Abstract

Chagas disease is a neglected endemic disease prevalent in Latin American countries, affecting around 8 million people. The first-line treatment, benznidazole (BNZ), is effective in the acute stage of the disease but has limited efficacy in the chronic stage, possibly because current treatment regimens do not eradicate transiently dormant *Trypanosoma cruzi* amastigotes. Nanostructured lipid carriers (NLC) appear to be a promising approach for delivering pharmaceutical active ingredients as they can have a positive impact on bioavailability by modifying the absorption, distribution, and elimination of the drug. In this study, BNZ was successfully loaded into nanocarriers composed of myristyl myristate/Crodamol oil/poloxamer 188 prepared by ultrasonication. A stable NLC formulation was obtained, with ≈80% encapsulation efficiency (%EE) and a biphasic drug release profile with an initial burst release followed by a prolonged phase. The hydrodynamic average diameter and zeta potential of NLC obtained by dynamic light scattering were approximately 150 nm and −13 mV, respectively, while spherical and well-distributed nanoparticles were observed by transmission electron microscopy. Fourier-transform infrared spectroscopy, differential scanning calorimetry, thermogravimetric analysis, and small-angle X-ray scattering analyses of the nanoparticles indicated that BNZ might be dispersed in the nanoparticle matrix in an amorphous state. The mean size, zeta potential, polydispersity index, and %EE of the formulation remained stable for at least six months. The hemolytic effect of the nanoparticles was insignificant compared to that of the positive lysis control. The nanoparticle formulation exhibited similar performance in vitro against *T. cruzi* compared to free BNZ. No formulation-related cytotoxic effects were observed on either Vero or CHO cells. Moreover, BNZ showed a 50% reduction in CHO cell viability at 125 µg/mL, whereas NLC-BNZ and non-loaded NLC did not exert a significant effect on cell viability at the same concentration. These results show potential for the development of new nanomedicines against *T. cruzi*.

## Introduction

Chagas disease is a neglected disease endemic to Latin America, affecting around 8 million people and causing 2000 deaths per year, according to the World Health Organization [1]. Currently, this health problem is not restricted to Latin American countries, as it has spread to non-endemic regions such as the United States and Europe [2–3]. It is caused by the hemoflagellate protozoan *Trypanosoma cruzi*, whose life cycle involves transitioning from non-flagellated multiplicative intracellular forms (amastigotes) to blood-circulating non-multiplicative forms (trypomastigotes). It is mainly transmitted by an insect vector of the Triatominae subfamily, although other modes of transmission (blood transfusion, organ transplant, and congenital transmission) have gained importance over the last decades. It is characterized by two stages: acute, and chronic. During the acute stage, which lasts up to two months after infection, the patients might present or mild, nonspecific, or no symptoms. This phase is followed by a chronic stage where parasites can be primarily found inside specific tissues. Decades after infection, signs and symptoms of damage to target organs, mainly the heart, gastrointestinal tract, and brain appear in 20–30% of chronically infected individuals [1,4].

Currently, two drugs have been approved for the treatment of Chagas disease: benznidazole (BNZ) and nifurtimox. The first-line treatment, BNZ, is a nitroimidazole that generates radical intermediates via the reduction of its nitro group, which covalently bind to macromolecules under aerobic and anaerobic conditions [5]. Cure rates are high when BNZ is administered during the acute phase [6]; however, in the chronic stage the cure rate is estimated to be less than 10% [7]. Some authors differ about this percentage owing to the variability in sensibility of the tests that are used to establish cure criteria [8–9]. BNZ is associated with a variety of adverse reactions including allergic dermatitis, hypersensitivity syndrome, gastric pain, anorexia, insomnia, vomiting, which ultimately lead to withdrawal in 12–18% of the patients [10]. Additionally, the BENEFIT (Benznidazole Evaluation for Interrupting Trypanosomiasis) trial could not prove that the standard treatment with BNZ can prevent disease progression [11].

BNZ has been classified as a class IV drug (low solubility, low permeability) in the Biopharmaceutics Classification System (BCS) [12]. It has an apparent volume of distribution (*V*_d_) of 0.56 L/kg, and reactive products of its metabolism [13]. Such *V*_d_ and low permeability values across biological barriers could result in difficulties for BNZ to reach intracellular amastigotes. The encapsulation of BNZ within nanoscale pharmaceutical carriers has been proposed as a strategy to reduce toxicity and improve efficacy [13]. Incorporation of drugs into nanoscale vehicles could result in changes in its absorption, distribution, metabolism, and excretion, which in turn could translate into improved efficacy and diminished BNZ toxicity. For example, BNZ-loaded nanoparticles could accumulate in the site of inflammation delivering the drug in the surroundings of their molecular target. In addition, nanocarriers may pass through the cell membrane via endocytosis to avoid BNZ efflux via the P-glycoprotein efflux pump [14–16], thus delivering the drug more efficiently. Many developments have been made in the past years resulting in lipid formulations such as liposomes, solid lipid nanoparticles (SLNs), and nanoemulsions, which increased the apparent solubility of BNZ and its efficacy against parasites [17]. Remarkably, oil-in-water nanoemulsions improved the trypanocidal activity against trypomastigotes compared to that of the free drug [18]. Among the aforementioned nanosystems, SLNs have recently gained special attention owing to their biocompatibility properties, biodegradability, relatively easy surface and composition modification, and efficacy in loading and delivering active principles [19]. SLNs comprise a lipid core, solid at 25 °C, stabilized by steric effects with a surfactant. The addition of small amounts of a liquid lipid at 25 °C leads to the improvement of SLNs in terms of sustained drug release and encapsulation efficiency (EE%), enabling the development of nanostructured lipid carriers (NLC) [20].

Here, we resort to NLC encapsulating BNZ, describing the preparation, physicochemical and biopharmaceutical characterization, and in vitro evaluation against *T. cruzi* intracellular and blood circulating forms. Interestingly, our formulation achieves a higher cumulative release and considerable higher activity against amastigotes compared to previously reported BNZ-loaded NLCs. Moreover, we report the dose-response intrinsic activity of myristyl myristate, a relatively common constituent of NLCs, against *T. cruzi*, which might be of future interest to other researchers working in the field.

## Results and Discussion

### Formulation and physicochemical characterization of NLC-BNZ

**Figure 1:**
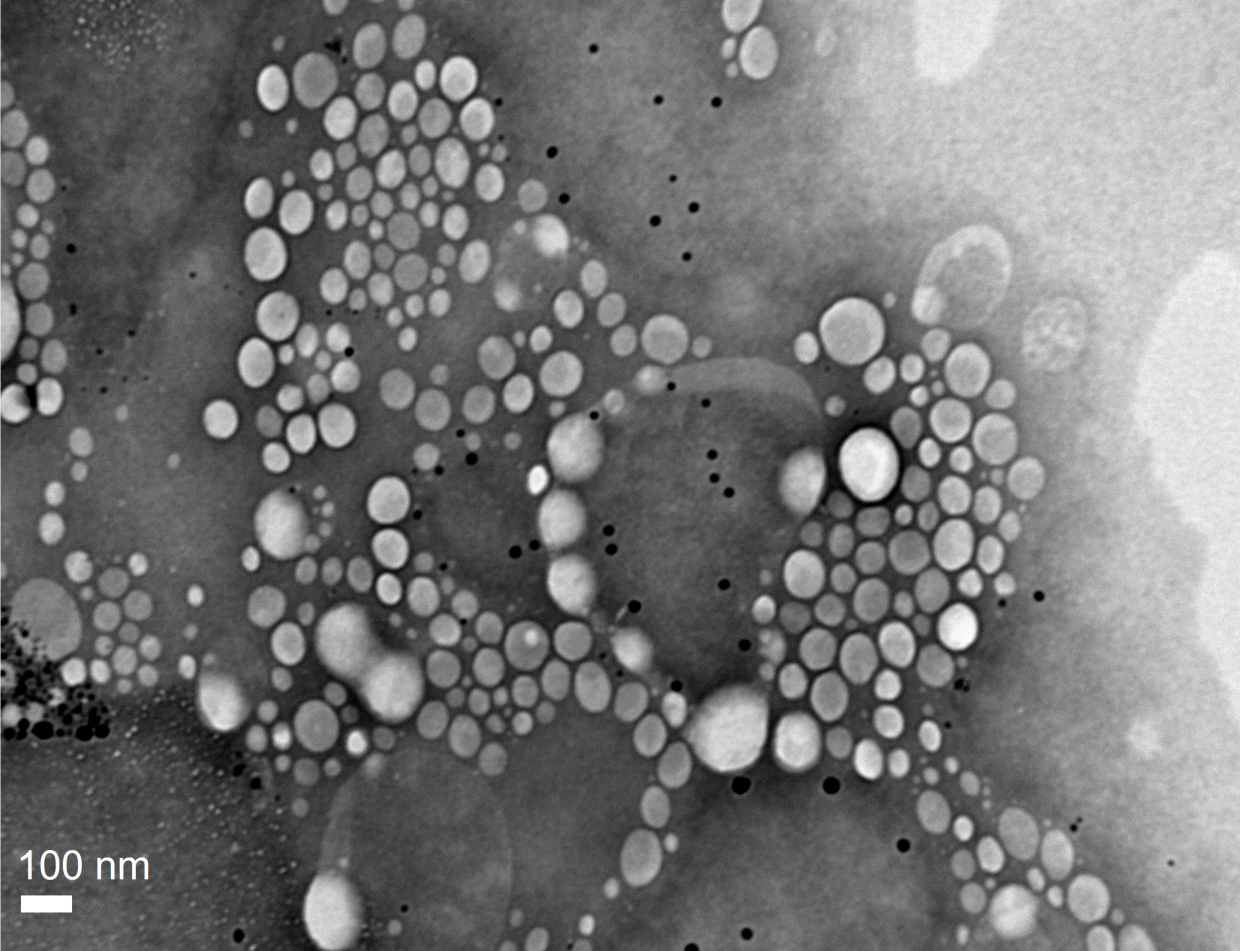
TEM image of NLC-BNZ.

Nanoparticle formulations were prepared by the ultrasonication method and named as NLC-BNZ or NLC-VEHICLE, in that order, depending on whether they contained BNZ or not. Stable homogeneous formulations were prepared. The encapsulation efficiency of NLC-BNZ was considerably high for the lipid formulations, reaching approx. 80%. The theoretical drug loading was 2.5%. Our results were in concordance with the encapsulation results of a previous study by Vinuesa et al., involving different types of nanoparticles and BNZ, including SLN and NLC [21]. The NLC-BNZ formulation was analyzed using transmission electron microscopy (TEM) to confirm the presence of nanoparticles showing a spherical morphology and a narrow distribution of sizes (Figure 1). Image analysis through ImageJ [22] software showed a mean particle size of 150 ± 13 nm.

Accordingly, the hydrodynamic diameter of the nanoparticles measured by dynamic light scattering (DLS) was in the 100–200 nm range (≈150 nm), with a moderate distribution of sizes as indicated by a polydispersity index (PdI) of 0.204. The zeta potential (ζ) was measured by Doppler anemometry, and it was found to be around −13 mV.

**Figure 2:**
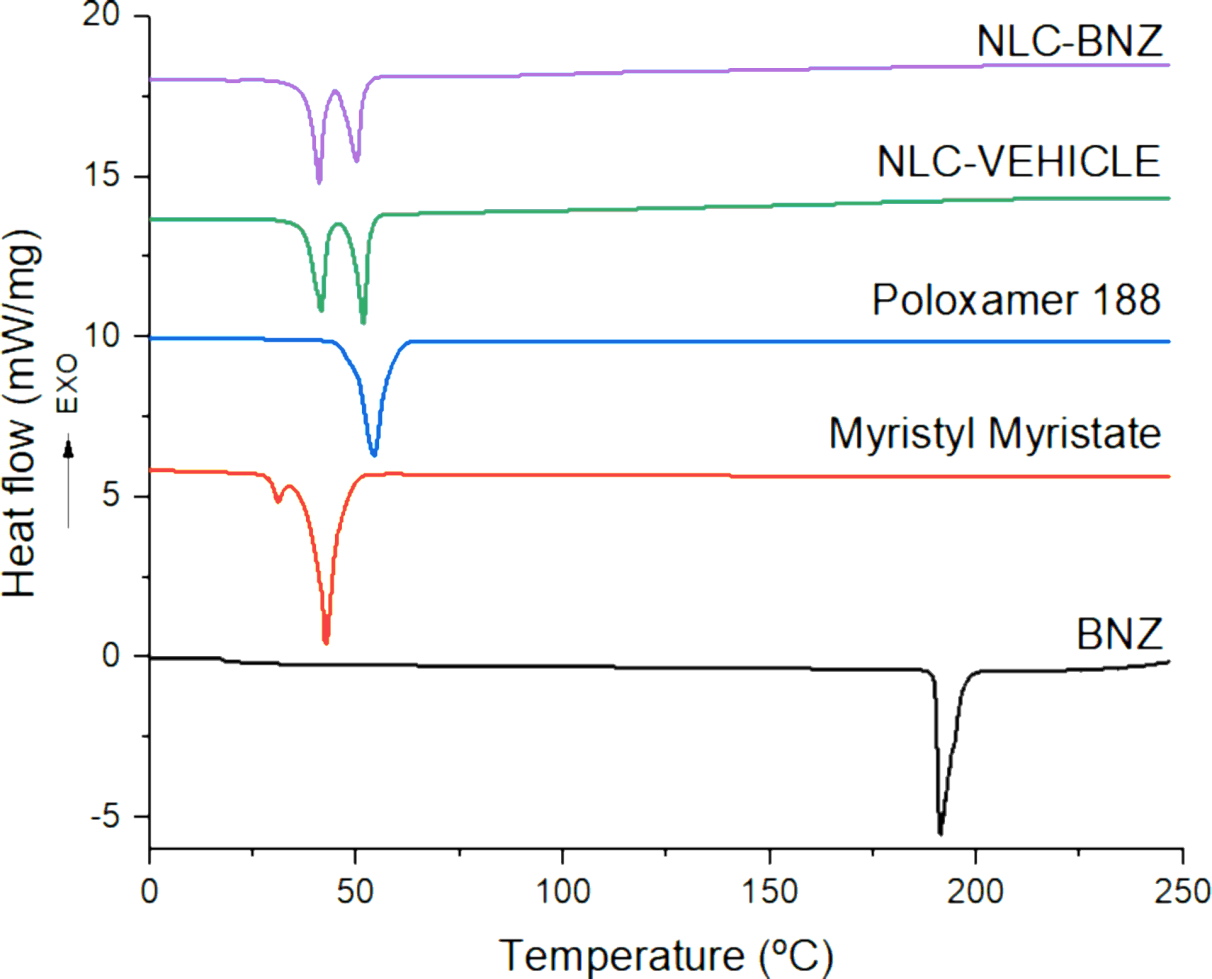
DSC thermograms of BNZ, myristyl myristate, poloxamer 188, NLC VEHICLE, and NLC-BNZ.

Differential scanning calorimetry (DSC) and thermogravimetric analysis (TGA) were performed to determine the thermal stability and melting/recrystallization processes of the components after drug encapsulation. Overlaid DSC thermograms are shown in Figure 2, whereas the melting temperature (*T*_m_), the enthalpy of fusion (Δ*H*_f_), and crystallinity index (CI) are presented in Table 1. Whereas BNZ showed an endothermic peak at its melting point (191.2 °C) [23], the formulation showed two endothermic peaks in the range of 40–50 °C, which could be referred to the melting points of the lipid and the surfactant, respectively. This suggests that no other endothermic changes occur to the formulation constituents or its load during the high-energy sonication procedure. A peak matching the phase transition peak of BNZ did not appear in the nanoparticle thermogram, indicating that BNZ was dispersed within the lipid matrix [24]. Correlating with the lower enthalpy of fusion, the CI (%) value of the nanoparticles was lower than that of the bulk myristyl myristate. Lipid molecules could be less ordered in the nanoparticles than in the bulk material, considering the disarrangement caused by the incorporation of the drug and the surfactant. For that reason, it might require less energy to melt in comparison to the pure crystalline substance [25].

**Table 1:** Thermal properties of benznidazole (BNZ), myristyl myristate, poloxamer 188, and nanoparticles (NLC-VEHICLE and NLC-BNZ). Abbreviations: *T*_m_, melting temperature; Δ*H*_f_, fusion enthalpy; CI (%), crystallinity index.

Sample	*T*_m_ (°C)	Δ*H*_f_ (j/g)	CI (%)

BNZ	191.2	142.3	100
myristyl myristate	42.9	239.4	100
poloxamer 188	54.2	148.8	100
NLC-VEHICLE	41.4–51.7	61.1	12.7
NLC-BNZ	40.9–50.0	59.4	12.4

**Figure 3:**
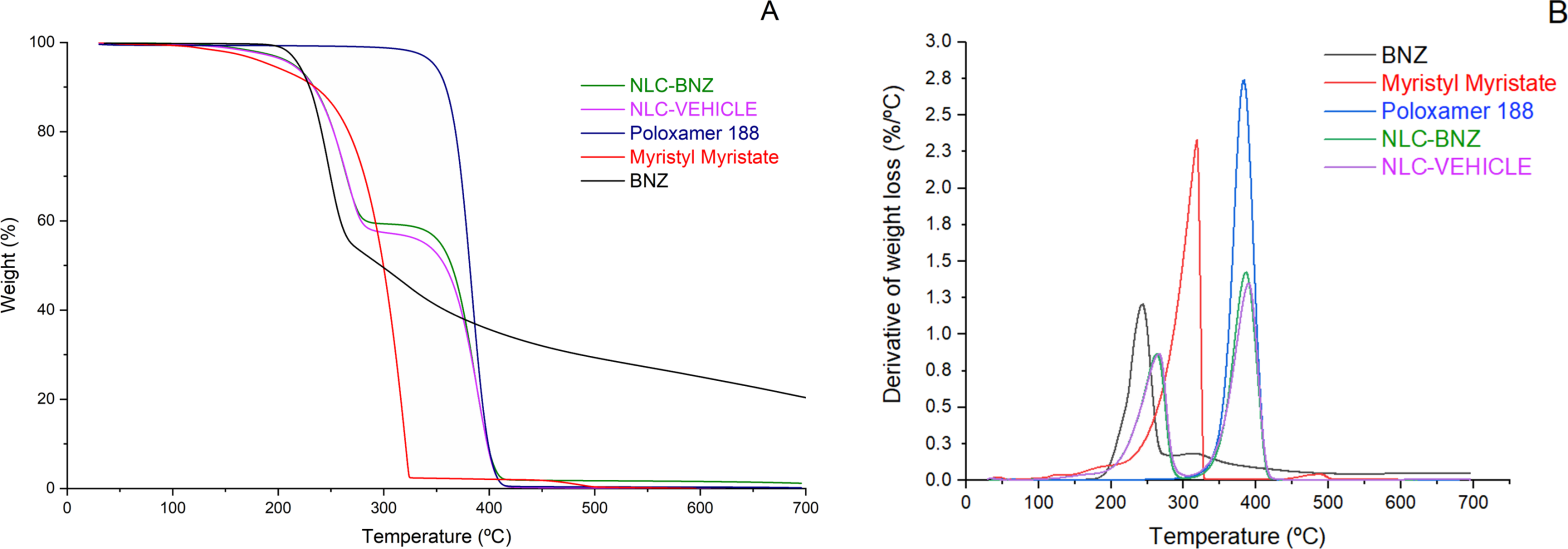
Thermogravimetric (A) and derivative thermogravimetric (B) curves of BNZ, myristyl myristate, poloxamer 188, NLC-VEHICLE, and NLC-BNZ.

Thermogravimetric curves of myristyl myristate, poloxamer 188, and BNZ showed one thermal degradation process, whereas NLC-BNZ and NLC-VEHICLE presented two events (Figure 3). That was also observed in the derivative curves. The weight loss process for the lipid started at 180 °C and finished at 320 °C. The poloxamer 188 thermogram showed a decomposition process starting at 300 °C and ending at 410 °C, and BNZ degradation occurred in the 190–300 °C range. Considering these processes, nanoparticle thermal behavior might be attributed first to lipid degradation, and second to poloxamer weight loss.

**Figure 4:**
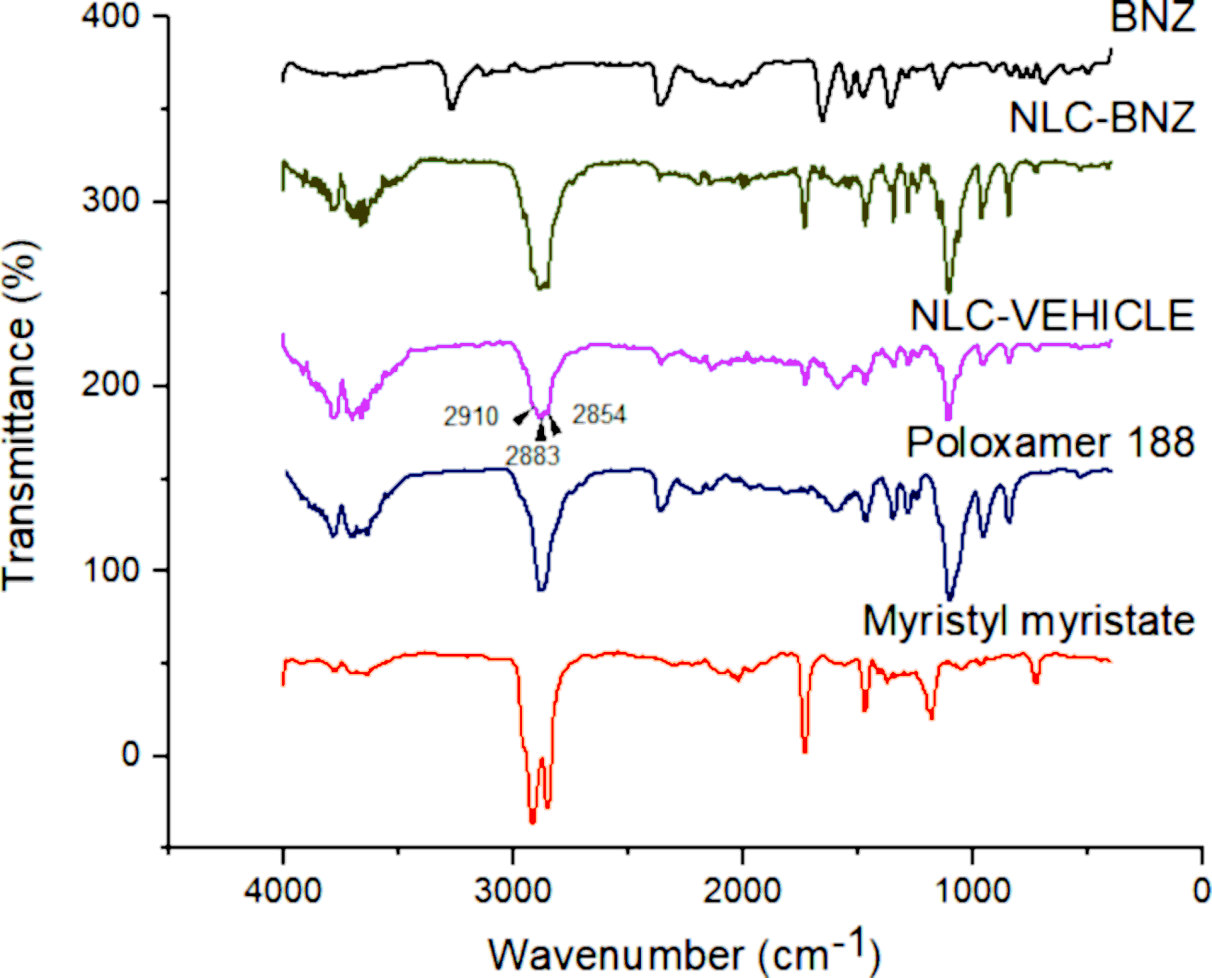
ATR-FTIR spectra of BNZ, myristyl myristate, poloxamer 188, NLC VEHICLE, and NLC-BNZ.

The attenuated total reflection Fourier-transform infrared spectroscopy (ATR-FTIR) technique was used to analyze the nanoparticle surface composition and determine the possible interactions among the formulation components (Figure 4). The BNZ spectrum presented its characteristic peaks at 3264 cm^−1^ corresponding to N–H in the secondary amide bond, 1652 cm^−1^ to C=O in the amide, 1523–1400 cm^−1^ to N–H flexion in the amide (1500–1400 cm^−1^ is also the absorption range of the C=C in the benzyl group), 1357 cm^−1^ to the symmetric vibration of R–NO_2_, and 1141 cm^−1^ to C–N in the imidazole ring [26]. Myristyl myristate displayed peaks at 2913 and 2848 cm^−1^ corresponding to C–H of alkane, 1731–1184 cm^−1^ to C=O and C–O stretching of ester groups, respectively. The peak at 1467 cm^−1^ was associated with the deforming vibrations of the C–H of alkanes [27]. The characteristic peaks of poloxamer 188 were at 3600 cm^−1^ relative to the O–H stretching, the intense peak at 2873 cm^−1^ corresponding to C–H stretching of alkanes, another intense peak at 1105 cm^−1^ to the symmetric stretching of C–O–C, and 964–833 cm^−1^ to asymmetrical and symmetrical stretching of C–C–O, respectively [28]. The NLC-BNZ spectra showed myristyl myristate and poloxamer characteristic peaks (overlapping of the most intense peaks in the 3000 cm^−1^ region – 2910 cm^−1^, 2883 cm^−1^, and 2854 cm^−1^ – due to the presence of the lipid and surfactant). In contrast, the spectra did not show peaks that could be linked to BNZ, suggesting that drug molecules were not on the nanoparticle surface but rather dispersed into the lipid matrix [24].

**Figure 5:**
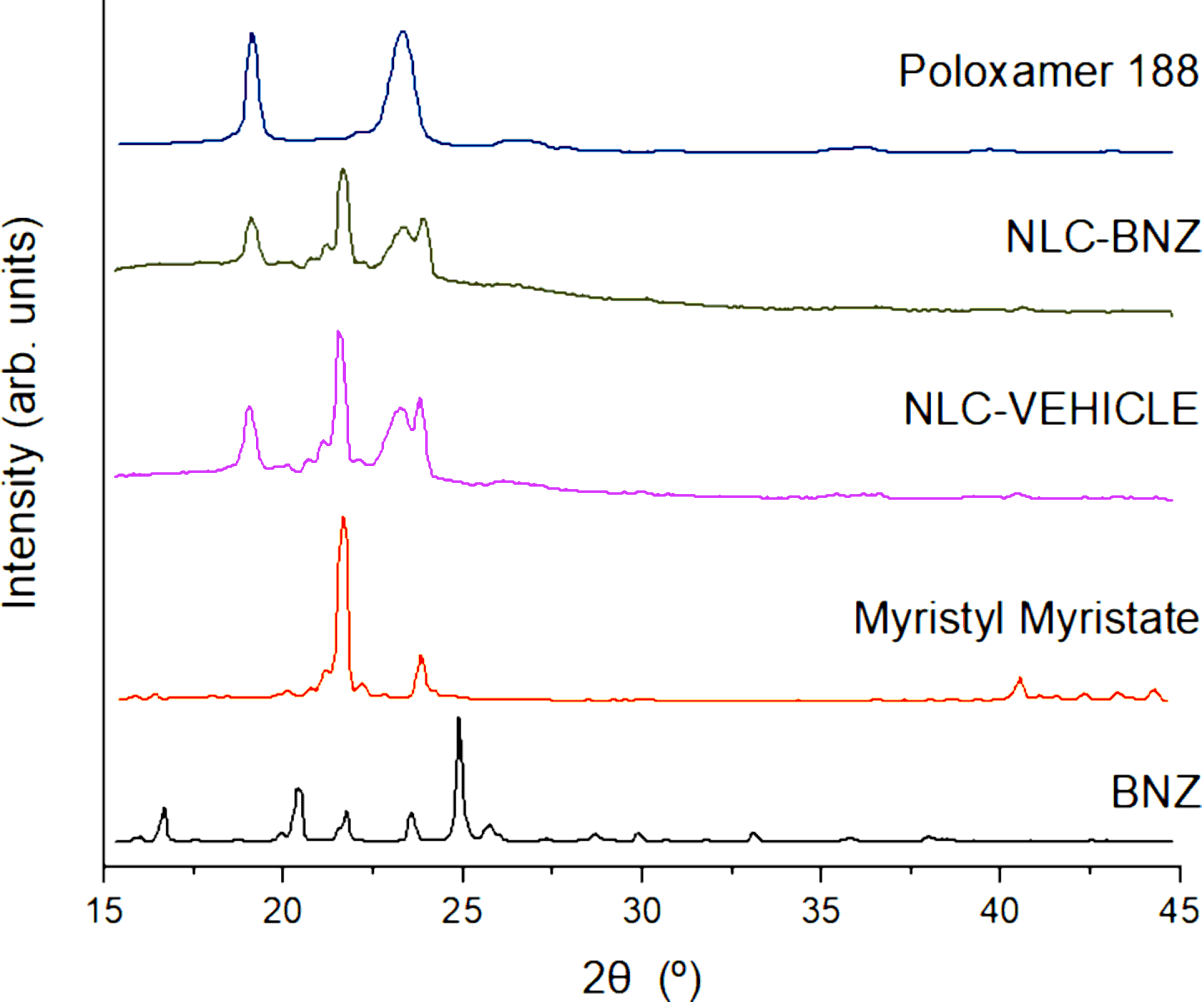
WAXS patterns for BNZ, myristyl myristate, poloxamer 188, NLC-VEHICLE, and NLC-BNZ.

Structural analysis was performed by selecting different angular regions from the small-angle X-ray scattering (SAXS) and wide-angle X-ray scattering (WAXS) patterns. The WAXS patterns (Figure 5) showed contributions of diffraction peaks from BNZ, myristyl myristate, and NLC. The nanostructured lipid carriers showed contributions from both the isolated myristyl myristate and additional Bragg peaks at 19.1° and 23.3° corresponding to the copolymer. This indicates that there was phase segregation, most likely a core–shell structure with the lipidic phase inside and the hydrophilic part of the copolymer in the outer part of the NLC. Myristyl myristate major peak positions expressed in terms of *d*_spacing_ were 4.1 and 3.8 Å, corresponding to a family of the β’ polymorph [29] and did not change after NLC synthesis or BNZ addition. Furthermore, there were no contributions from the crystalline phase of BNZ within NLC because of its small quantity or to dissolution inside the NLC.

**Figure 6:**
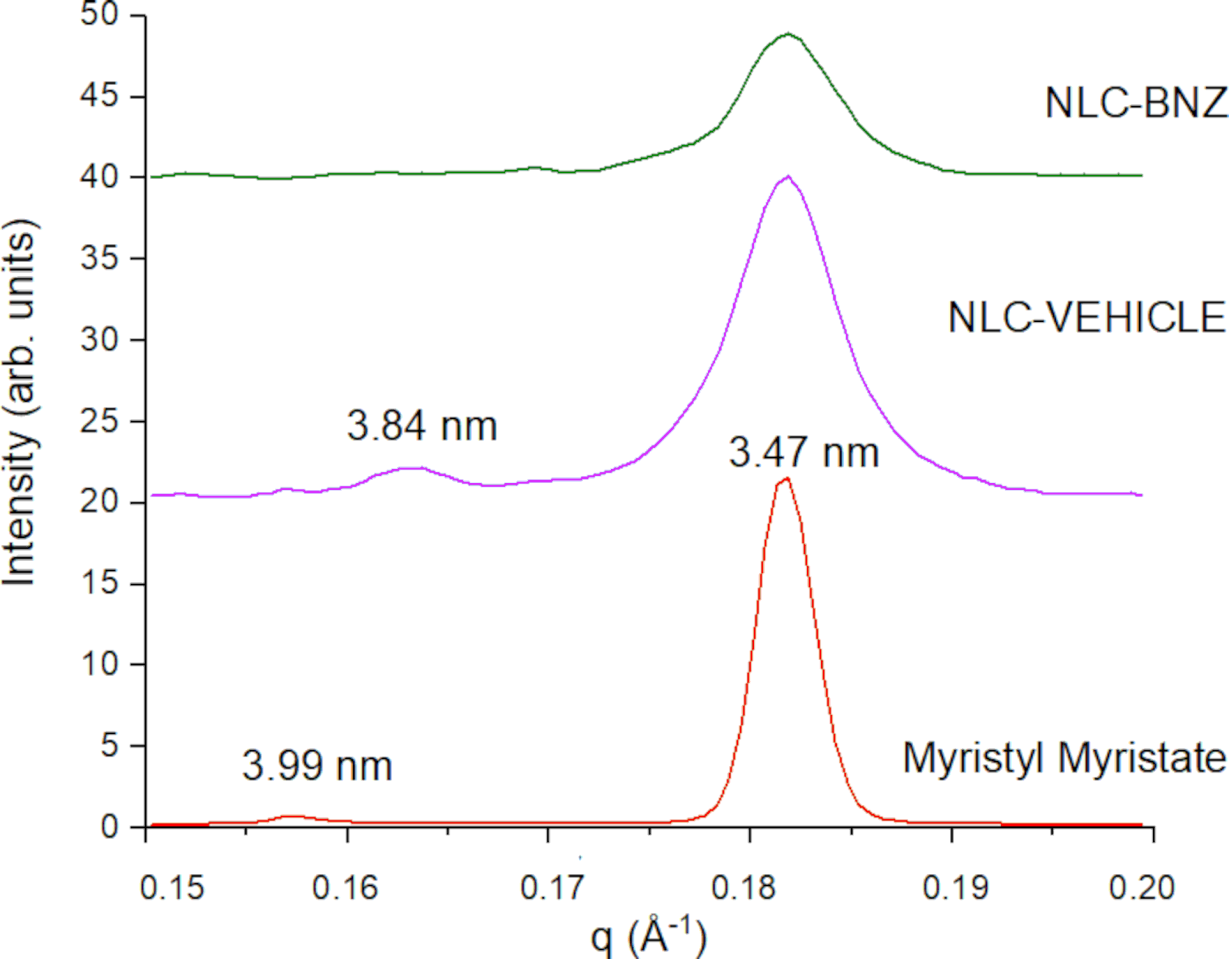
SAXS patterns for samples with myristyl myristate in their composition.

**Figure 7:**
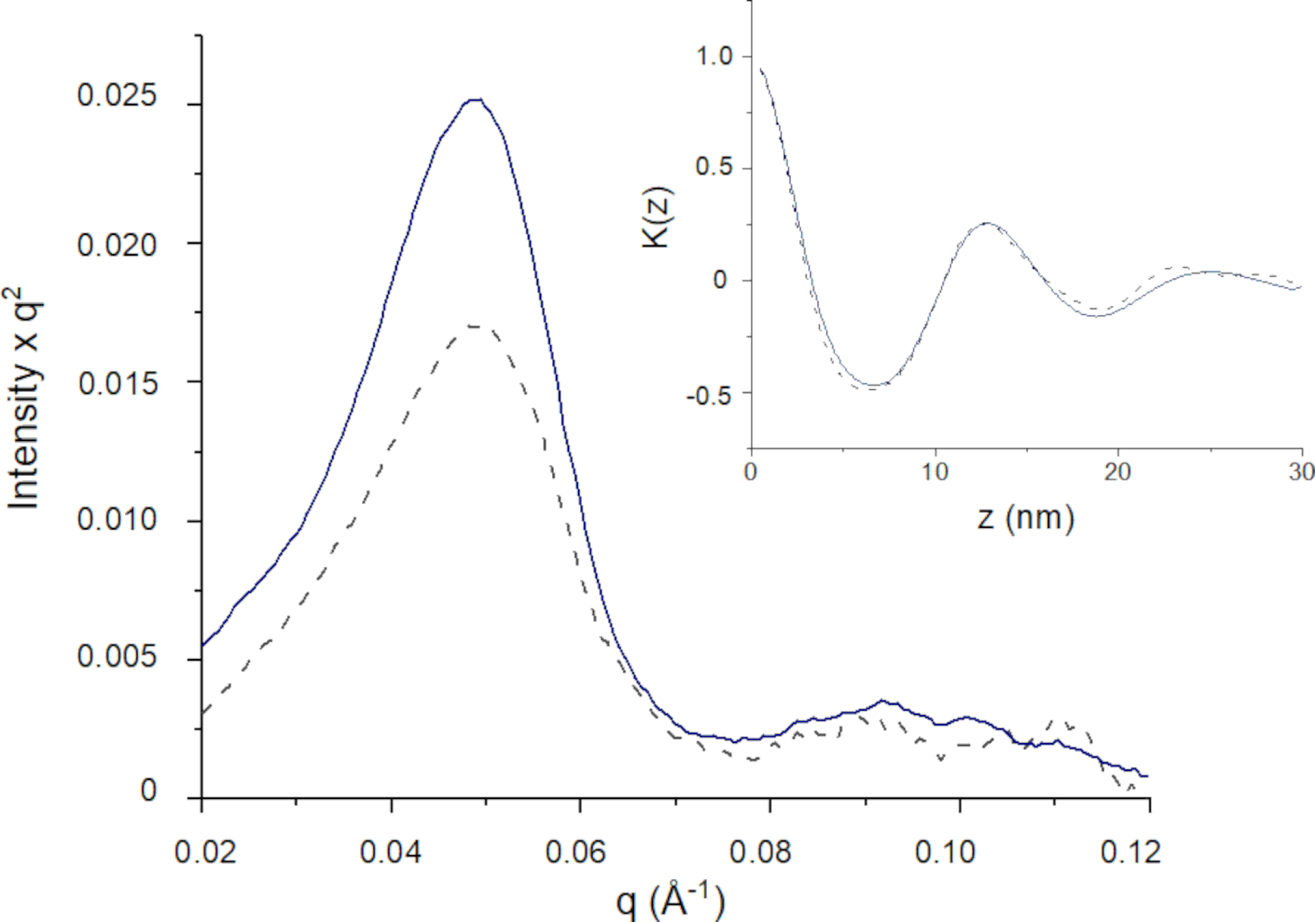
Lorentz/Kratky plot for SAXS patterns of NLC-VEHICLE (continuous line) and NLC-BNZ (dashed line). The inset corresponds to the linear correlation transformation for lamellar systems.

The long period Bragg diffraction peaks for Myristyl myristate could be observed in SAXS patterns at the *q* range between 0.15 and 0.2 Å^−1^ (Figure 6). Bare myristyl myristate confirmed the presence of a β’ polymorph with long period *d*_spacing_ of 3.99 (001) and 3.47 nm (002), while in the NLC only the 3.47 nm of *d*_spacing_ peak remains. Also, in the NLC systems, the main Bragg peak was wider, attributed to a nanosized crystal effect where the estimated crystallite average sizes were 94 ± 5 nm and 101 ± 5 nm for NLC-VEHICLE and NLC-BNZ, respectively, using the Scherrer approximation. However, a broadening of the lower part of the main peak in the NLC-BNZ samples suggests defects in the structure, probably due to the inclusion of BNZ in the formulation. At smaller angles, the copolymer on the surface exhibited a lamellar-like structure [30]. The Lorentz/Kratky plot (*q*²*I* vs *q*) is shown in Figure 7, where the peaks of NLC and NLC-BNZ remained at the same position, independently of the presence of the BNZ load. The linear correlation function was obtained by using the following transformation (Equation 1) [31–32]:


[1] $$K\left(z\right)=\frac{\int_{0}^{\infty }I_{\text{norm}}\left(q\right)q^{2}\cos\left(qz\right)\text{d}q}{\int_{0}^{\infty }I_{\text{norm}}\left(q\right)q^{2}\text{d}q}$$

where *I*_norm_ is the normalized intensity after removing the myristyl myristate (i.e., MM) contribution: *I*_norm_ = *I*_NLC_(*q*) – *I*_background_ – *c*(*I*_MM_(*q*) – *I*_background_), *c* being a constant or weighted proportionality between phases. From this transformation the lamellar period obtained from the first maximum of the oscillation was 12.6 nm for both systems (Figure 7). In contrast with amphiphilic low-weight loading, BNZ is a lipophilic molecule that did not change the structure of the copolymer. Thus, it is proposed to be dissolved in the core of the lipidic nanoparticle.

### Drug release and physical stability

**Figure 8:**
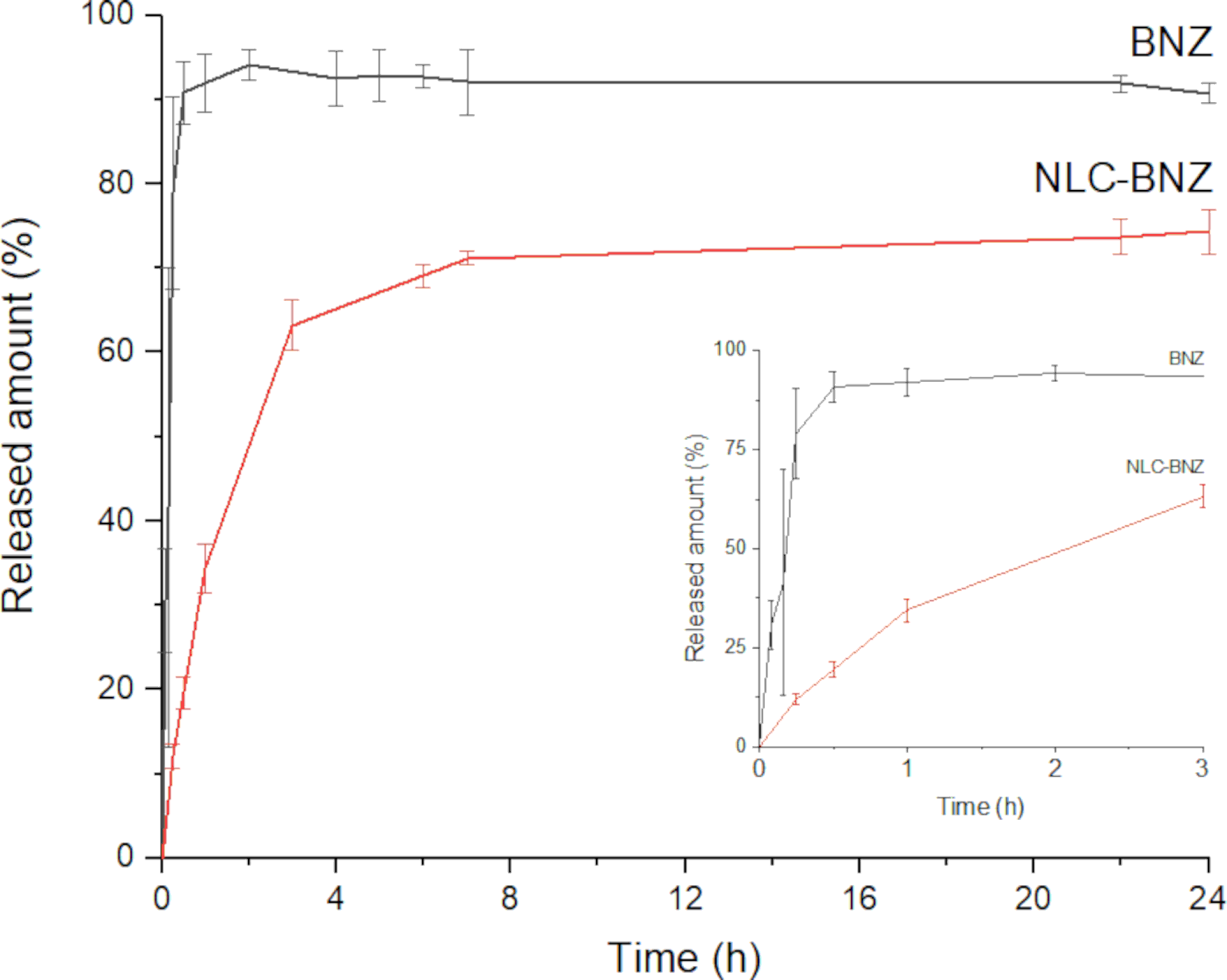
In vitro release profile of free BNZ and NLC-BNZ for 24 hours. The inset graph shows the release profiles during the first three hours.

The release profiles (Figure 8) showed that 78% of the free drug was dissolved in the first 15 min of the experiment. In contrast, during the first 15 min only about 12% of the drug was released from the NLC formulation. An initial burst release was observed, followed by a sustained release. This phenomenon could be explained in part by considering the presence of free drug molecules in the formulation (around 20% of the initial drug load) and in part by the release of drug molecules located near the surface of the NLC, which rapidly diffuse out of the vehicle. The slow increase of the drug concentration in the release medium observed after the initial stage could be attributed to the gradual release of drug molecules from the matrix core, where the drug is mainly located according to X-ray diffraction (XRD) results [33]. Remarkably, although our NLC possess a comparatively lower drug load, the maximal accumulated drug release is higher than that of similar systems previously reported [21].

The in vitro release data were fitted to different mathematical models. The model that best adjusted the data was the Korsmeyer–Peppas model followed by a first-order model (Supporting Information File 1, Table S1). The Korsmeyer–Peppas model, also called power law, was initially used to describe drug release in polymeric systems where the two predominant mechanisms were relaxation of the polymer chains and diffusion. In this model (Equation 2), *M_t_*/*M*_∞_ is the fraction dissolved, *K* is a constant that incorporates structural and geometrical information, and the exponent *n* is the diffusional or transport exponent, that provides information about the release mechanism. However, it can also be viewed as a generalization for the explanation of two different drug release mechanisms that could coexist [34]. The mechanism that dominates the release can be inferred through the value of the release exponent *n*. For spherical systems, *n* will take a value of 0.43 for drug release governed by Fickian diffusion, a value of 1 for zero-order release, and intermediate values for intermediate behavior, often regarded as anomalous transport. In our case, the estimated value of *n* was 0.56, suggesting mixed release mechanisms at play with a strong contribution of diffusion. As in our case there is no polymer relaxation involved, it may be hypothesized that the burst effect could be slightly affecting the global kinetics of the process [35]. Although this description has its limitations, it has been widely used to describe drug release from similar lipidic formulations [35–38].


[2] $$\frac{M_{t}}{M_{\infty }}=Kt^{n}$$

**Figure 9:**
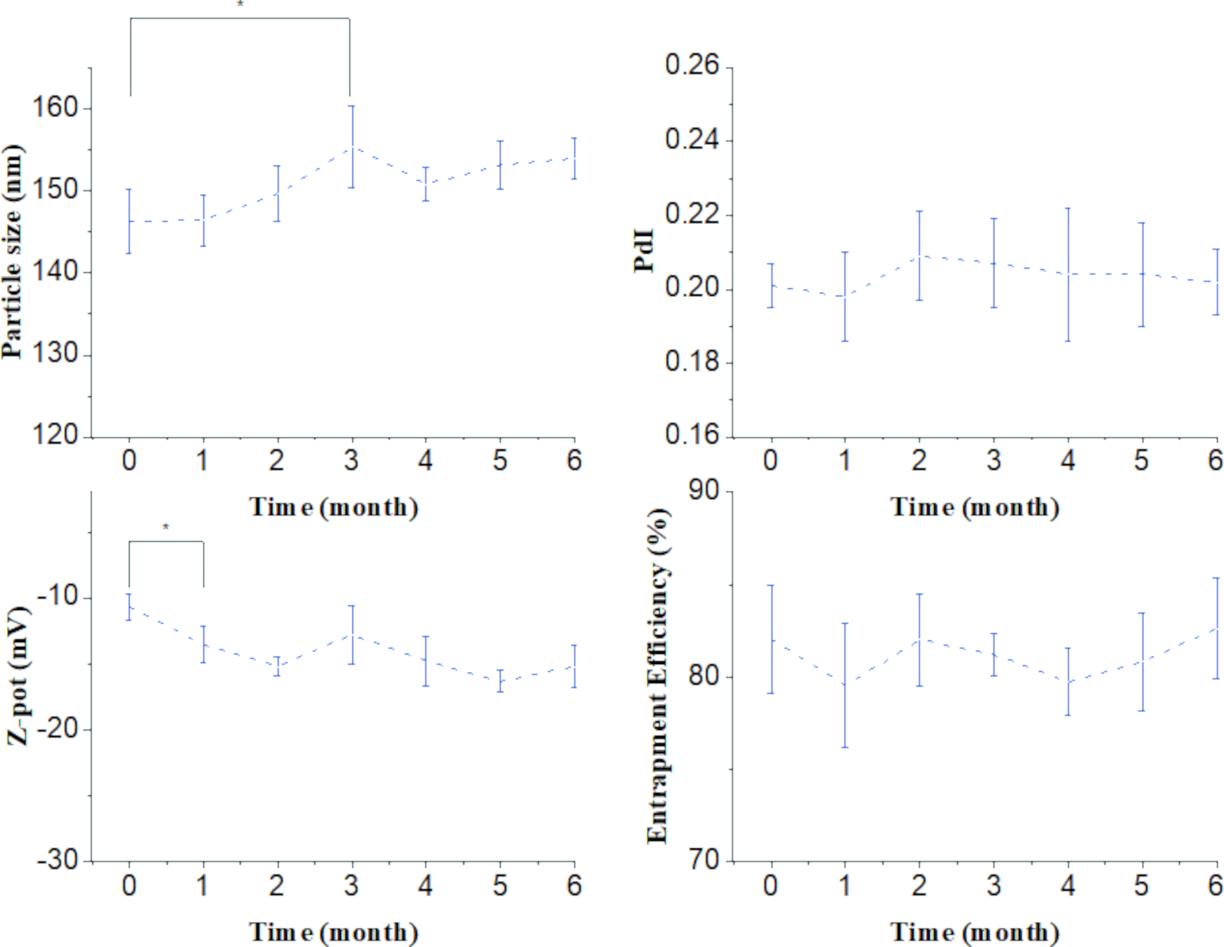
Followed-up of relevant physical parameters of the formulation for up to six months to test its stability under the selected storage conditions (refrigerator at 4 °C), * = *p* < 0.05.

The mean particle size, PdI, zeta potential, and encapsulation efficiency were selected as parameters to follow the physical stability of the nanoparticle dispersion for six months under the selected storage conditions (refrigerator at 4 °C) (Figure 9). Based on these results, the formulation could be stored at 4 °C for at least three months without losing its initial properties in terms of size; polydispersity and encapsulation efficiency values remained unaltered during the storage period, and the zeta potential parameter started at −10 mV and ended up at −15 mV after six months. Dynamic light scattering analysis of the formulations revealed nanoparticles with a hydrodynamic diameter in the 100−200 nm range (≈50 nm) starting at 146 nm and slightly increasing in the third month up to 155 nm. The Z-average parameter was chosen to report the nanoparticles size. The size values were consistent with the TEM image analysis. The zeta potential (ζ) was measured by Doppler anemometry and operated as a report of the formulation surface characteristics. The surface charges required to achieve a good dispersion of nanoparticles stabilized by electrostatic repulsion are around ±30 mV [39]. The ζ value of our formulation was ≈14 mV. Although this value is not optimal for stabilization by electrostatic repulsion, it still contributes with a positive aspect, as high-negative ζ values may impede cellular uptake [40]. On the other hand, it was observed that the nanoparticulated systems remained stable after six months with no precipitation. This suggests that in this case the stabilization is not achieved by means of surface charge alone, but also by the steric repulsion after adding a non-ionic surfactant [41].

### Cytotoxicity and hemolytic activity

**Figure 10:**
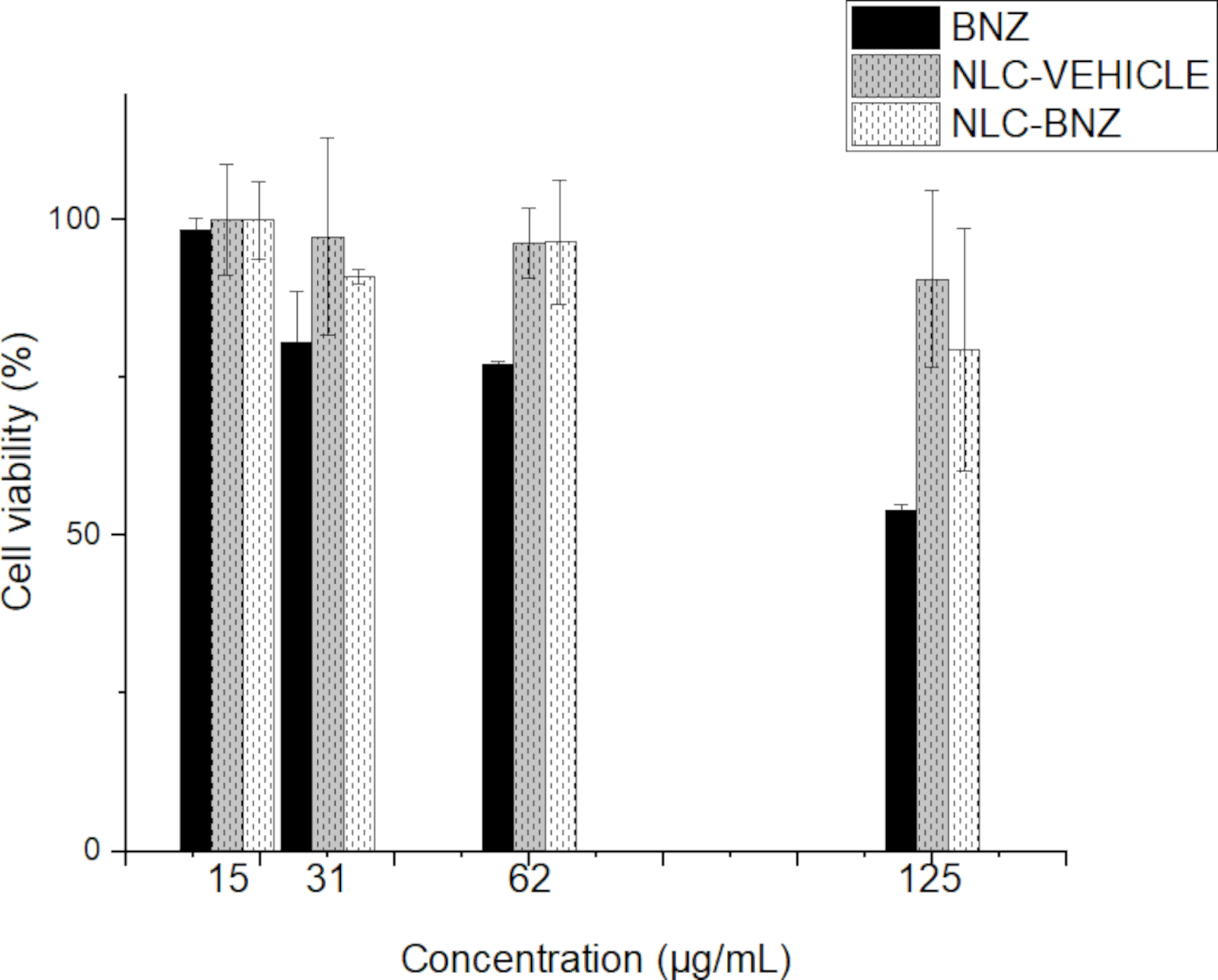
Cell cytotoxicity in CHO cells treated with free BNZ, NLC-VEHICLE, and NLC-BNZ.

Cytotoxicity assays using the tetrazolium 3-[4,5-dimethylthiazol-2-yl]-2,5-diphenyltetrazolium bromide salt method (MTT) showed that Chinese hamster ovary cells (CHO) viability was affected by BNZ concentration in a dose-dependent manner (Figure 10). Interestingly, the cell viability for NLC-VEHICLE or NLC-BNZ at the same tested concentrations of free BNZ resulted in values above 80% in all cases, suggesting a decreased cytotoxic effect. That decrease in toxicity generated by NLC-BNZ, in comparison with free BNZ, could be attributed to the release profile of BNZ from NLC, exposing cells to lower doses of BNZ during the first stages of cellular division. This is a remarkable result, as toxic effects of BZN are a major cause of treatment discontinuation in the clinical setting [42]. Additionally, cytotoxicity was evaluated in the Vero cell line by flow cytometry, where the percentage of dead cells labeled with propidium iodide (PI, Supporting Information File 1, Figure S2) was measured. Neither the drug-loaded or unloaded NLCs elicited significant toxicity in Vero cells.

**Figure 11:**
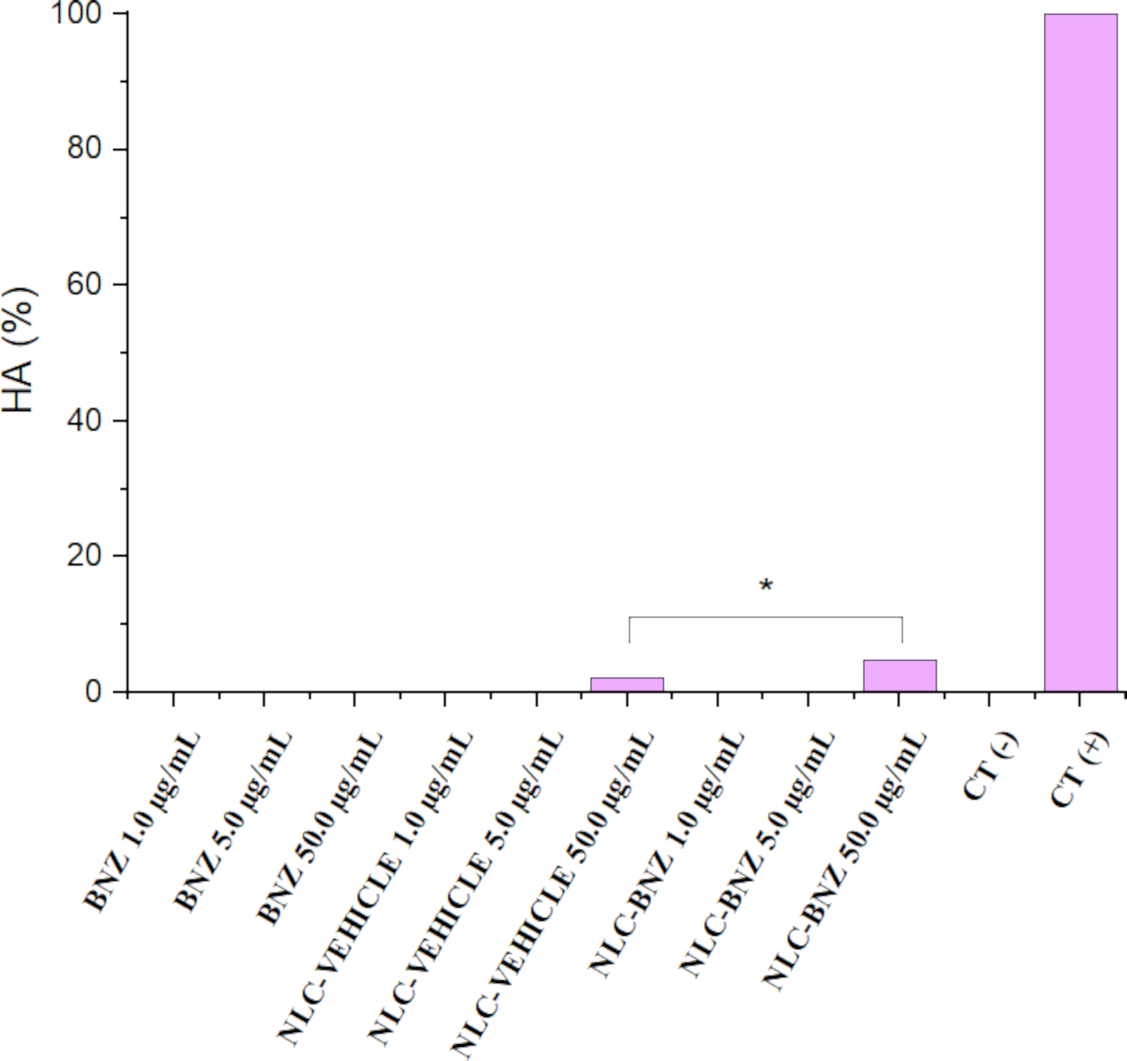
Hemolytic activity (%) of BNZ, NLC-VEHICLE, and NLC-BNZ at three different concentrations. * = *p* < 0.05.

As it is common to parenterally administer nanoparticle formulations, it is of interest to study the potential toxicity of pharmaceutical nanocarriers in blood cells. Most of the published papers evaluated the hemolytic activity (HA) of nanoparticles after 2, 3, or 5 h of incubation [43–45]. The standard methods to test hemolytic activity of nanoparticles (ISO/TR 7406 or ASTM E2524-08 standard) established that biomaterials that induce a critical hemolytic ratio of <5% can be considered safe for biological applications [46]. In our study, it was observed no hemolytic effects for BNZ, NLC-VEHICLE, and NLC-BNZ at different concentrations after 3 and 24 h of incubation (data not shown). However, some hemolytic activity was observed for NLC-VEHICLE and NLC-BNZ after 48 h of incubation (Figure 11). Despite NLC-BNZ showed 4.8% HA at the highest concentration, the formulation could still be considered safe according to the regulations. In fact, hemolytic activity could be caused by several reasons, including the ageing of the blood sample after 48 h of incubation with the concomitant release of hemoglobin, but also by the presence of surfactants that could destabilized the erythrocyte membrane [47]. On the other hand, the differences between NLC-VEHICLE and NLC-BNZ, the latter exhibiting a higher HA, could be explained by adding the HA of the free drug to the effect of the vehicle on erythrocytes. More studies would be necessary to investigate the effect of the composition, size, or porosity of these nanoparticles after a long term exposure to blood samples as was described for other type of nanoparticles [45]. Our results suggest that the reported NLC-BNZ formulations are hemocompatible [43].

### In vitro antiparasitic activity

**Figure 12:**
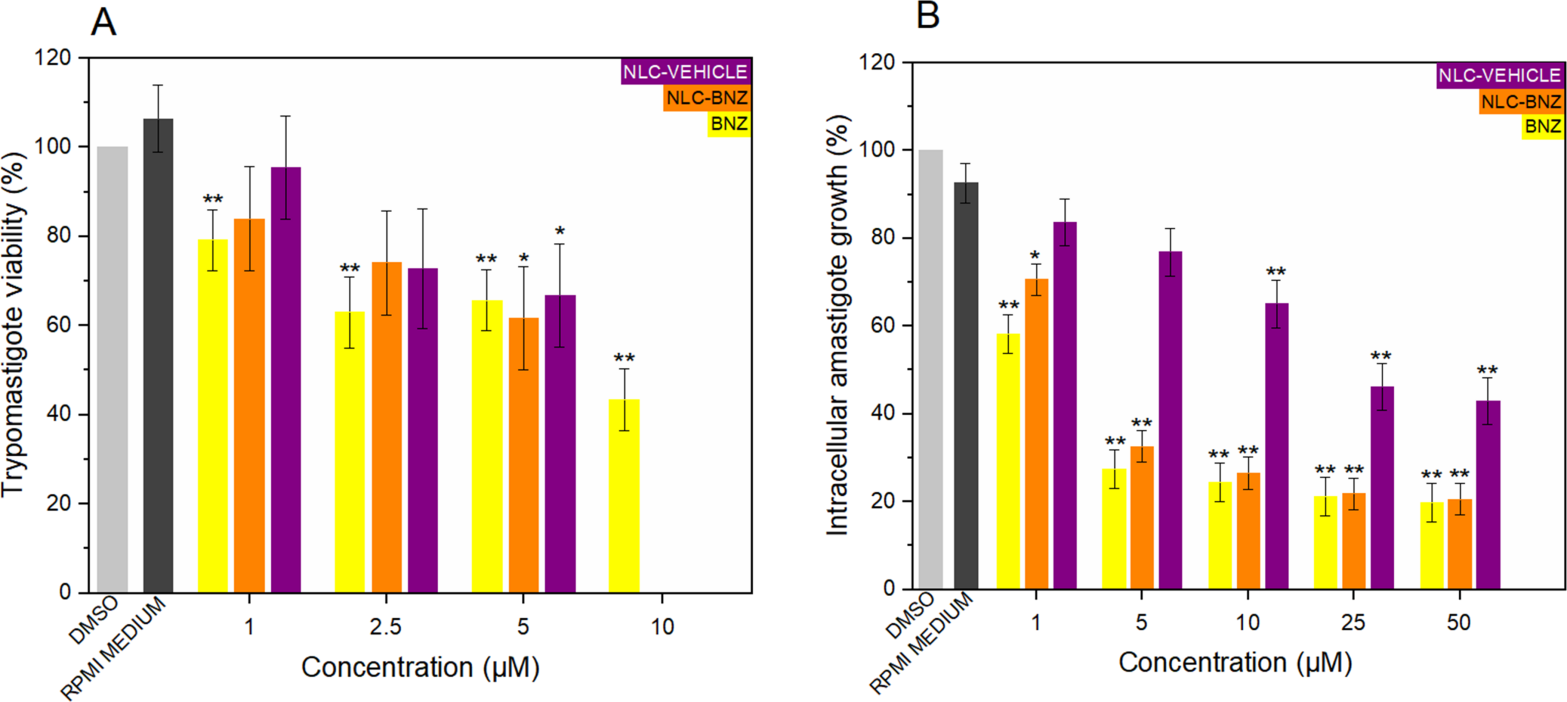
Dose-response effect of BNZ, NLC-BNZ, and NLC-VEHICLE (empty, non-loaded with BNZ) on *T. cruzi* trypomastigotes (A) and amastigotes (B). For NLC-VEHICLE, the assayed concentrations of empty NLC correspond to those that would contain the indicated concentration of BNZ. Dunnet’s test was performed to identify significant differences against the control group. *p < 0.05, **p < 0.01.

As shown in Figure 12A, free BNZ displayed a clear dose-dependent effect on *T. cruzi* trypomastigotes (with an EC_50_ of 6.07 µM), whereas the NLC-BNZ and NLC-VEHICLE also exhibited a dose-response behavior despite comparatively large variability across replicates. While for free BNZ the estimated EC_50_ value was 6.07 µM with similar reported values (6.04 µM [48]) for the same parasite classification (TcI), the NLC-BNZ presented a full trypanocidal effect at concentrations higher than 5 µM (10 µM). A similar observation was found for the empty particles (NLC-VEHICLE) suggesting that the formulation itself possesses intrinsic toxicity on *T. cruzi* trypomastigotes. A separate assay of the individual constituents of the formulations was thus performed, demonstrating that myristyl myristate, at a relatively low concentration, has a negative effect on parasite viability (dose-response studies for myristyl myristate against amastigotes are shown in Supporting Information File 1). This may imply that myristyl myristate cannot be considered, in our case, as a pharmacologically inert constituent in our formulation. Instead, it should be considered as a pharmaceutical active ingredient based on its intrinsic effects against *T. cruzi*.

The dose-response effects of BNZ, NLC-VEHICLE, and NLC-BNZ on *T. cruzi* amastigotes were also evaluated (Figure 12B), and the corresponding EC_50_ were calculated. Benznidazole and NLC-BNZ presented inhibition of the intracellular growth of the parasites even at the lowest concentration, with no significant differences observed between the treatments. Benznidazole and NLC-BNZ EC_50_ values were 3.15 and 3.33 µM, respectively. In agreement with the in vitro trypanocidal assay, NLC-VEHICLE also displayed intrinsic anti-amastigote activity with an EC_50_ value of 10.29 µM. This was unexpected, although not necessarily a negative outcome, having in mind that our formulation displayed reduced cytotoxicity against mammal cells. Isolated myristyl myristate lipid was also tested against amastigotes, showing a reduced amastigote density at effective concentrations (Supporting Information File 1, Figure S1). Neither P188 nor GTCC-LQ displayed any effect against trypomastigotes or amastigotes up to 50 µM. Of note, the more efficacious NLC encapsulating BNZ previously reported in the literature [21] had an EC_50_ against amastigotes of 17.6 µM. The higher efficacy of our system may be explained by a higher maximal drug release and/or by the intrinsic activity of myristyl myristate, which adds to that of BNZ.

A hypothesis about the intrinsic toxicity of our nanoscale vehicle on *T. cruzi* may be linked to a modification of glycosylphosphatidylinositols (GPIs). Glycosylphosphatidylinositols are the main anchor complexes used by protozoans to bind to cell surface proteins. It covalently attaches to the C terminus of a protein connecting it to the outer leaflet of the lipid bilayer [49]. *Trypanosoma brucei* predominant membrane protein variant surface glycoprotein (VSG), which is involved in parasite host immune system evasion, is anchored by a GPI that requires myristate for its synthesis. Analogs of myristate have shown toxicity towards the parasite [50]. *T. cruzi* trypomastigotes connect mucin (a surface molecule implicated in parasite virulence) to the membrane through a GPI which is synthesized exclusively with a C16 fatty acid [51], though a C14 fatty acid incorporation could be toxic to the parasite. Experiments in *T. brucei* indicated that the specificity of fatty acid incorporation depends on chain length [52]. The lipid in our formulation is an ester of fatty acids that could hypothetically interrupt the anchoring of mucin to the lipid bilayer in *T. cruzi,* thus rendering the parasites non-viable. However, further studies are required to test this hypothesis.

## Conclusion

Among the spectra of nanoformulations encapsulating BNZ that exist to date, the nanoparticles presented in this work might be considered a novelty in terms of the lipid and manufacturing technique of choice. We achieve physical stability for at least six months with acceptable particle size, PdI, and EE%. Complementary to these results, TEM images showed a spherical configuration. Thermal and crystallographic experiments indicated that BNZ was dispersed into the lipid matrix. The formulations showed a sustained drug release profile for 24 h, achieving a maximal accumulated release above 74% during 24 h. The release profile was adequately fitted to the Korsmeyer–Peppas model with an estimated release exponent of 0.56, suggesting a mixed mechanism of release with a dominant Fickian behavior. In vitro experiments on *T. cruzi* trypomastigotes and amastigotes showed similar performances against the intracellular form of the parasite when comparing encapsulated and free BNZ. Surprisingly, the empty nanoparticles exhibited activity against the parasite, which was later attributed to one of the constituents of the formulation, myristyl myristate. This may explain why our formulations exhibited increased performance against *T. cruzi* compared with other previously reported BNZ-loaded NLC. It would be interesting to study the effect of other lipids on the parasite to optimize the efficacy of the formulations based on a potential additive or synergistic effect of BNZ and the formulation itself. Remarkably, the cytotoxicity effect on host cells was lower for the BNZ-loaded nanoparticles compared to that of the free drug, showing a possible benefit for the use of our formulation.

## Experimental

### Materials

Benznidazole (Lot #MKCD5602, purity ≥ 97%) and Kolliphor^®^P188 (poloxamer 188) were purchased from Sigma-Aldrich. Myristyl myristate (Crodamol™ MM, melting range = 36–40 °C), and the oil (Crodamol^TM^ GTCC-LQ, a mixture of fully saturated triglycerides, melting point = −5 °C) were kindly donated by Croda Argentina. All reagents used in the preparation and analysis of the formulations were of analytical grade and were obtained from different commercially available sources.

### Formulation of benznidazole-loaded nanostructured lipid carriers

BNZ-loaded NLCs were obtained via ultrasonication as previously described in Scioli-Montoto et al. (2022) [53]. Solid myristyl myristate (2% w/v, 400 mg) was melted in a water bath at 60–70 °C. The oil (40 μL) was added to the melted lipid phase simultaneously with BNZ (10 mg). The aqueous phase was prepared by dissolving 600 mg (3% w/v) of poloxamer 188 (poly(ethylene glycol)-block-poly(propylene glycol)block-poly(ethylene glycol)) in 20 mL of ultrapure water (Milli-Q^®^, Millipore, Ma., USA) and was preheated at the same temperature as the melted lipid in the water bath. After 30 min of thermostatization, the aqueous solution was poured over the lipid phase, and ultrasonication was carried out for 20 min at an 80% amplitude using an ultrasonic processor (130 Watts, Cole-Parmer, USA) equipped with a 6 mm titanium tip. After the sonication process, NLC-BNZ were obtained by leaving the hot suspension to cool to room temperature. The remaining volume was then measured.

### Measurement of the encapsulation efficiency

Concentration of the free drug in the dispersion medium was measured to calculate the encapsulation efficiency (EE%). For this, 500 μL of the formulation was placed in Microcon^®^ centrifugation filters (MWCO = 10000, Merck Millipore, Billerica, MA, USA) and centrifuged at 10000 rpm for 15 min. The amount of BNZ was estimated by performing a high-performance liquid chromatography (HPLC) analysis of the filtrate. Considering the initial amount of BNZ added to the formulation, the EE% was calculated as follows:


[3] $$EE\left(\%\right)=\frac{M_{0}-\left(C_{\text{free}}\cdot V_{f}\right)}{M_{0}}\times 100$$

where *M*_0_ is the initial amount of BNZ added to the formulation, *C*_free_ is the drug concentration of the filtrate (i.e., the free drug concentration) in μg/mL, and *V*_f_ is the volume after ultrasonication (mL).

The theoretical drug loading (DL%) was calculated as follows:


[4] $$\text{DL}\left(\%\right)=\frac{\text{Mass of drug incorporated }\left(\text{mg}\right)}{\text{Lipid mass }\left(\text{mg}\right)}\times 100$$

### HPLC analysis of benznidazole

Chromatographic separation was achieved by HPLC (Gilson SAS, Villiers-Le-Bel, France) via UV detection. A Platinum EPS C8 (150 mm × 4.6 mm, 5 μm, Grace^TM^, Columbia, MD, USA) column was used; the mobile phase consisted of a mixture of methanol and 0.02% phosphoric acid solution (60:40) for a final pH of 2.5. The system was operated isocratically at a 1.0 mL/min flow rate and the detection was performed at 324 nm. The volume of injection was 20 μL.

### In vitro benznidazole release assay

The release of BNZ from the nanoparticles was performed in a rotating paddle apparatus (Vision Classic 6, Hanson Research, Chatsworth, CA, USA) at 75 rpm using 500 mL of KH_2_PO_4_ buffer (pH 6.8) as the dissolution medium. The bath temperature was set at 37.0 ± 0.5 °C. A volume of 5 mL of each formulation was placed in a pre-hydrated dialysis membrane (MWCO 10 kDa) and submerged into the dissolution vessels. A solution of free BNZ at the same concentration was used as control. At 0, 5, 10, 15, and 30 min, and at 1, 2, 3, 4, 5, 6, 7, and 24 h, 1 mL of the dissolution medium was taken from the vessel. Samples were analyzed by HPLC as described above. Experiments were performed in triplicate and the mean values were used for data analysis. The data were fitted to mathematical models of drug release (i.e., First order, Hopfenberg, Baker–Lonsdale, Korsmeyer–Peppas, and Hixon Crowell) via the DDSolver complement developed by Zhang et al. and available for Excel^®^ [54]. The model that best fitted the data according to the goodness-of-fit measures (*R*^2^, *R*^2^-adj, MSE, and AIC) was chosen.

### Particle size, zeta potential and polydispersion index

Nano ZS Zetasizer (Malvern Instruments Corp, Worcestershire, UK) was used to measure particle size distribution and mean diameter by DLS at 25 °C in polystyrene cuvettes with a thickness of 10 mm. The zeta potential was determined by Doppler anemometry using the previously described equipment. As an estimation of the distribution of particle sizes, the polydispersion index was determined. All experiments were carried out in triplicate, except for the particle size estimation, which was measured six times.

### Physical stability

The mean particle size, PdI, zeta potential, and encapsulation efficiency were measured to assess the physical stability of the nanoparticle dispersion during storage at 4 °C protected from light. Physical parameters (e.g., particle size, PdI, zeta potential) were measured by DLS and EE% was measured by HPLC, once a month, during a six-month period.

### Differential scanning calorimetry analysis

Thermal analysis of BNZ, myristyl myristate, poloxamer 188, and NLC-BNZ was performed by differential scanning calorimetry (DSC Q2000, TA Instruments, New Castle, DE, USA) under an inert atmosphere of dry nitrogen (50 mL·min^−1^). A standard aluminum pan containing approximately 5 mg of the dry sample after freeze drying the formulations was used. Scans were run in the range from 0 to 250 °C at a heating rate of 10 °C/min.

The degree of crystallinity (% crystallinity index, CI) was calculated using the following equation [55]:


[5] $$\text{CI}\left(\%\right)=\frac{\Delta H_{\text{NLC aqueous dispersion}}}{\Delta H_{\text{bulk material}}\times {\text{ Concentration}}_{\text{lipid phase}}}\times 100$$

where Δ*H*_NLC_ and Δ*H*_bulk material_ are the melting enthalpies (J·g^−1^) of the NLC dispersion and the bulk lipid, respectively. The concentration of the lipid phase was 2%.

### Thermogravimetric analysis

Thermogravimetric analysis was performed to assess the thermal stability of BNZ, myristyl myristate, poloxamer 188, and NLC-BNZ on a TGA Q500 apparatus (TA Instruments, New Castle, DE, USA). Freeze dried formulations of approximately 10 mg were accurately weighed in a platinum pan. Measurements were performed from room temperature to 600 °C at a heating rate of 10 °C/min under nitrogen atmosphere to avoid thermo-oxidative degradation.

### Attenuated total reflection Fourier-transform infrared spectroscopy

Fourier-transform infrared spectroscopy spectra were obtained. The attenuated total reflection mode was used to record the spectra over the range of 400–4000 cm^−1^ at a resolution of 2 cm^−1^.

### Transmission electron microscopy

Transmission electron microscopy images were captured using a Jeol-1200 EX II-TEM microscope (Jeol, MA, USA). A drop (10 µL) of the nanoparticle dispersion previously diluted (1:10) with ultrapure water was spread onto a collodion-coated Cu grid (400 mesh). Excess liquid was drained with filter paper. A drop of phosphotungstic acid was added to the dispersion for contrast enhancement.

### X-ray diffraction structural analysis

Small angle X-ray scattering/wide angle X-ray scattering measurements were performed using a XEUSS 2.0 equipment (XENOCS, France). Patterns were registered with two synchronous 2D photon-counting pixel X-ray detectors for SAXS Pilatus 200k (DECTRIS, Switzerland), and a Pilatus 100k (DECTRIS, Switzerland) placed 159 mm from the sample with a tilted angle of 36 °C for WAXS. The SAXS measurements were performed using two samples to detect distances, 1194 and 337 mm, to obtain a wide angular range. The scattering intensity, *I*(*q*), was recorded by means of the scattering momentum transfer *q*, where *q* = 4π/λ sin(θ), 2θ is the scattering angle and λ = 0.15419 nm is the weighted average of the X-ray wavelength of the Cu Kα_1,2_ emission lines. Owing to the small beam size pointed at the sample (< 1 mm × 1 mm) smearing effects were not considered. The NLC samples were placed under vacuum between Kapton^®^ tapes. The measurements were done in transmission mode. The SAXS/WAXS patterns were taken for 10 min each.

### Cell toxicity assay on CHO cells

The viability of CHO cells was analyzed by the reduction of the tetrazolium salt to a formazan product (i.e., the MTT method). A 96-well polystyrene microplate containing 1 × 10^4^ cells per well of CHO cells (obtained from the American Type Culture Collection, Manassas, VA, USA) were cultured in Ham’s F12 medium (Gibco BRL, Grand Island, NY, USA) supplemented with 10% fetal bovine serum (FBS, Notocor Laboratories, Cordoba, Argentina) and antibiotics (50 IU penicillin and 50 μg/mL streptomycin) (Bagó Laboratories, Buenos Aires, Argentina) in a humidified atmosphere with 5% CO_2_. After 24 h, the cells were incubated with increasing concentrations of RPMI as control, BNZ, NLC-VEHICLE, and NLC-BNZ (0, 15, 31, 62, and 125 μg·mL^−1^). The MTT reagent (5 mg·mL^−1^ in phosphate-buffered saline (PBS)) was then added for 3 h. Dimethyl sulfoxide (DMSO,100 µL per well) was added under agitation for 10 min to dissolve the MTT. The color was measured in a microplate reader (MultiskanTM GO spectrophotometer, Thermo Fisher Scientific) at 550 nm. The assays were performed in triplicate.

### Cell toxicity assay on Vero cells

Cell viability was analyzed by flow cytometry as described in the “In vitro anti-amastigote effect” section after adding PI to obtain the percentage of dead cells following the incubation with the formulation of nanoparticles or the free drug.

### Hemolytic effect

Hemolysis was assessed on 3 mL of a freshly drawn heparinized suspension of fresh human blood placed on a 6-well cell culture plate. Increasing concentrations of freshly prepared dilutions of the free drug and NLC-BNZ (1, 5, and 50 μg·mL^−1^) were added to each well and incubated for 3, 24, and 48 h at 37 °C. Samples were then centrifuged for 5 minutes at 2500 rpm, and the absorbance of the supernatant was determined at 540 nm in a microplate reader (MultiskanTM GO spectrophotometer, Thermo Fisher Scientific). Triton X 100 (10%), saline solution, NLC-VEHICLE, and BNZ were used as the positive, negative, vehicle, and reference drug controls, respectively. The hemolytic activity was calculated as [56]:


[6] $$\text{HA}\left(\%\right)=\frac{A_{\text{540 nm}}\text{sample}-A_{\text{540 nm}}\text{saline}}{A_{\text{540 nm}}\text{Triton}-A_{\text{540 nm}}\text{saline}}\times 100$$

where *A*_540 nm_sample represents the absorbance value of the sample, *A*_540 nm_Triton the absorbance value of the positive control, and *A*_540 nm_saline the absorbance value of the negative control.

The blood was obtained from the “Institute of Hemotherapy” in La Plata, Buenos Aires, Argentina as part of a formal agreement between the “Instituto de Genética Veterinaria (IGEVET, UNLP-CONICET La Plata)” and this institution. Also, this assay protocol was approved by the National University of La Plata Ethics Committee and it was developed in accordance with the principles proclaimed in the Universal Declaration of Human Rights of 1948, the ethical norms established by the Nuremberg Code of 1947, and the Declaration of Helsinki of 1964 and its successive amendments and clarifications. Special attention was paid to Patient Rights in their relationship with health professionals and institutions and the National Law 25326 on the Protection of Personal Data.

### Parasites

The *T. cruzi* strain K98 (TcI, low virulence) was used. Tissue culture trypomastigotes were obtained from the supernatants of 2- to 3-day-old infected Vero cells (African green monkey kidney epithelial cells) maintained in RPMI-1640 medium supplemented with 10% FBS (Internegocios S.A, Argentina) at 37 °C in a 5% humidified CO_2_ atmosphere. Amastigotes were obtained after infecting Vero cells at a multiplicity of infection (MOI) of 1:2.

### In vitro anti-trypomastigote effect

A trypomastigote suspension (1 × 10^5^ trypomastigotes per well) was co-cultured in a 96 well-plate with dilutions of both a solution of the free drug and of the nanoparticle formulations (concentration range = 1, 2.5, 5, and 10 µM) in RPMI-1640 supplemented with 5% FBS at 37 °C in 5% CO_2_ atmosphere. The NLC-VEHICLE sample was tested using the same dilutions as the NLC-BNZ formulation. After 24 h of incubation, motile parasites were counted in a hemocytometer chamber under a light microscope. Controls consisted of RPMI-1640 supplemented with 5% FBS as well as RPMI-1640 with 0.1% of DMSO.

Results were expressed as mean viability of trypomastigotes (%) (regarding to RPMI-1640 + DMSO control). Experiments were performed in triplicate. The half maximal effective concentration (EC_50_) against the trypomastigote form was determined from concentration-response curves fitted through a nonlinear regression on GraphPad Prism version 8.0.1 software (San Diego, CA, USA).

### In vitro anti-amastigote effect

Vero cells were infected with the trypomastigote form of GFP-expressing *T. cruzi* (K98 strain) [57] at a multiplicity of infection (MOI) 1:2. After 24 h the cells were washed with PBS, trypsinized for 10 min, and seeded onto 96-well plates (5 × 10^4^ cells/well). After the cells attached to the microplate (i.e., 2–3 h), increasing concentrations of freshly prepared dilutions of the formulations (1, 5, 10, 25, and 50 µM) or the free drug were added. After 72 h of treatment, the cells were harvested with a trypsin/EDTA solution and processed for flow cytometry analysis using a BD Biosciences FACSCANTO II Flow Cytometer (Franklin Lakes, NJ, USA). Propidium iodide (Sigma, St. Louis, USA) was added to the cell suspensions (50 µg/mL) for 10 min, prior to analysis. In total, 20000 events were acquired for each sample. Data analysis was performed using the FlowJo^TM^ software (FlowJo, LCC). The EC_50_ values were determined from dose-response curves fitted through a non-linear regression using GraphPad Prism version 8.0.1 software (San Diego, CA, USA). The experiments were performed in duplicates.

### Statistical analysis

The normality of the variable distribution was assessed using the Shapiro–Wilk normality test. Comparisons of the means were performed by analysis of variance (ANOVA) followed by Tukey or Dunnet comparison tests. Statistical significance was set at *p* < 0.05.

## Supporting Information

This file includes a summary of the goodness-of-fit measures that indicate how different mathematical models of drug release fit our experimental data.

File 1 Supplementary material.

## Authors

Giuliana Muraca^1^, María Esperanza Ruiz^1^, Rocío C. Gambaro^2^, Sebastián Scioli-Montoto^1^, María Laura Sbaraglini^1^, Gisel Padula^2^, José Sebastián Cisneros^3^, Cecilia Yamil Chain^3^, Vera Alvarez^4^, Cristián Huck-Iriart^5,6^, Guillermo Castro^7^, María Belén Piñero^8^, Matias Ildebrando Marchetto^8^, Catalina Alba Soto^8^, Germán A. Islan^9^* and Alan Talevi^1^*

## Addresses

^1^Laboratorio de Investigación y Desarrollo de Bioactivos (LIDeB), Departamento de Ciencias Biológicas, Facultad de Ciencias Exactas, Universidad Nacional de La Plata, La Plata, Argentina, ^2^Instituto de Genética Veterinaria (IGEVET, UNLP-CONICET La Plata), Facultad de Ciencias Veterinarias Universidad Nacional de La Plata (UNLP), La Plata, Argentina, ^3^Instituto de Investigaciones Fisicoquímicas Teóricas y Aplicadas (CONICET-UNLP), La Plata, Buenos Aires, Argentina, ^4^Grupo de Materiales Compuestos Termoplásticos (CoMP), Instituto de Investigaciones en Ciencia y Tecnología de Materiales (INTEMA), Facultad de Ingeniería, Universidad Nacional de Mar del Plata (UNMDP) – CONICET, Av. Colón 10850 (B7608FDQ), Mar del Plata, Buenos Aires, Argentina, ^5^Laboratorio de Cristalografía Aplicada, Escuela de Ciencia y Tecnología, CONICET, Universidad Nacional de San Martín (UNSAM), Campus Miguelete, 25 de Mayo y Francia, San Martín B1650KNA, Buenos Aires, Argentina, ^6^ALBA Synchrotron Light Source, Carrer de la Llum 2–26, Cerdanyola del Vallès, 08290 Barcelona, España, ^7^Nanomedicine Research Unit (Nanomed), Federal University of ABC (UFABC), Santo André, SP, Brazil, ^8^Instituto de Investigaciones en Microbiología y Parasitología Médica (IMPaM) - Consejo Nacional de Investigaciones Científicas y Técnicas - Universidad de Buenos Aires Departamento de Microbiología, Facultad de Medicina, Buenos Aires, Argentina and ^9^Centro de Investigación y Desarrollo en Fermentaciones Industriales (CINDEFI), Laboratorio de Nanobiomateriales, Departamento de Química, Facultad de Ciencias Exactas, Universidad Nacional de La Plata (UNLP)-CONICET (CCT La Plata), Calle 47 y 115, (B1900AJI), La Plata, Buenos Aires, Argentina
