# Supplementary material for: Nanostructured lipid carriers containing benznidazole: physicochemical, biopharmaceutical and cellular in vitro studies
Source: Beilstein J Nanotechnol. 2023 Jul 28;14:804–18. doi: 10.3762/bjnano.14.66 (PMC10390827; doi:10.3762/bjnano.14.66)
Supplement: File 1 — Supplementary material. [file Beilstein_J_Nanotechnol-14-804-s001.pdf]

BEILSTEIN JOURNAL OF NANOTECHNOLOGY

Supporting Information

for

Nanostructured lipid carriers containing benznidazole: physicochemical, biopharmaceutical and cellular in vitro studies

Giuliana Muraca, María Esperanza Ruiz, Rocío C. Gambaro, Sebastián Scioli-Montoto, María Laura Sbaraglini, Gisel Padula, José Sebastián Cisneros, Cecilia Yamil Chain, Vera A. Álvarez, Cristián Huck-Iriart, Guillermo R. Castro, María Belén Piñero, Matias Ildebrando Marchetto, Catalina Alba Soto, Germán A. Islan and Alan Talevi

Beilstein J. Nanotechnol. **2023,** *14,* 804–818. doi:10.3762/bjnano.14.66

Supplementary material

License and Terms: This is a supporting information file under the terms of the Creative Commons Attribution License (https://creativecommons.org/licenses/by/4.0). Please note that the reuse, redistribution and reproduction in particular requires that the author(s) and source are credited and that individual graphics may be subject to special legal provisions.
The license is subject to the *Beilstein Journal of Nanotechnology* terms and conditions: (https://www.beilstein-journals.org/bjnano/terms)

Table S1: Goodness-of-fit measures of mathematical models of drug release for NLC-BNZ.

Model	R^2	R^2-adjusted	MSE	AIC
First order	0.97	0.97	14	22
Hopfenberg	0.95	0.94	37	27
Baker–Lonsdale	0.90	0.90	57	29
Korsmeyer–Peppas	0.99	0.99	5	18
Hixon–Crowell	0.95	0.95	27	25

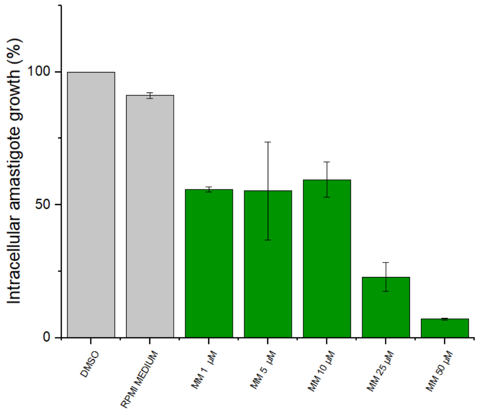

Figure S1: Dose-response effect of myristyl myristate on *T. cruzi* amastigotes.

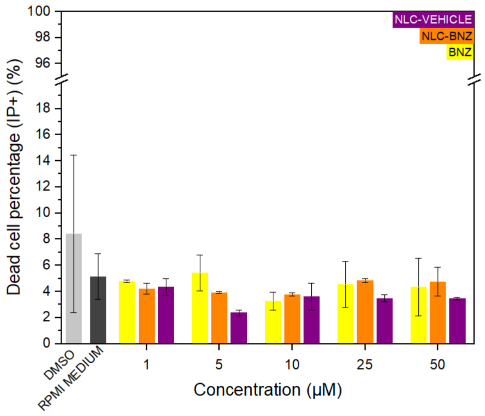

Figure S2: Flow cytometry measurements of cell cytotoxicity in Vero cells treated with free BNZ, NLC-VEHICLE, and NLC-BNZ.
